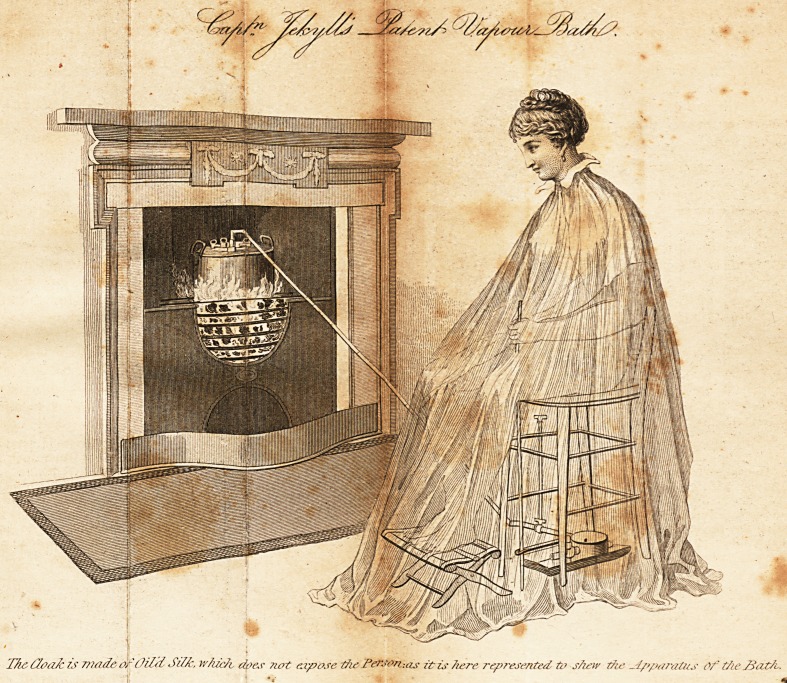# Medical and Physical Intelligence

**Published:** 1823-12

**Authors:** 


### MEDICAL AND PHYSICAL INTELLIGENCE.

### ANATOMY.

1. *Decussation of the Optic Nerves.*—VICQ-D'AZYR was the first who remarked, on examining the optic nerves of the human subject, which had been hardened by immersion in alcohol, that the medullary fibres of the external border of the superior and of the inferior surfaces of the decussation go immediately to the eye of the same side, but that the middle contains a homogeneous tissue. The WENZELS have come to the same conclusion, in examining with the microscope a horizontal section of the crossing of these nerves: they found the nerves composed of long fibres, some of which were large, and others again narrow, not manifestly separated from one another, and not straight, like muscular fibres, but variously arranged; that the greater part of these,—viz. that occupying the exterior side of the optic nerve,—follows the same direction from the eye, even to the optic thalamus; that, on the contrary, the smaller part, or that which is in the interior side of the nerve, is directed obliquely to the opposite side, without, however, any crossing of fibres being manifest at the point of junction. These observations are in a great measure confirmed by those of M. Treviranus, made, with the aid of the microscope, on the male *simia aygula*. The roots of the nerves and the brain were left during some months immersed in alcohol. A very moderate glass was sufficient to show the fibrous structure at the external border of these nerves. After they had been kept some time in caustic potash to soften them, having removed their sheath, and separated their fibres by means of a pincers and fine needles, he made out that the external fibres of the upper side of each was continued from their cerebral extremity to that in the eye, without uniting themselves to those of the other side; whilst, on the contrary, the internal and inferior fibres of one nerve went to the other side, and united with the fibres of the opposite nerve. It was difficult to decide positively whether any of the fibres actually passed from one side to the other: he thought that some did so. The total mass of the internal fibres interlaced together was perceptibly more bulky than that of the external, which ran to the eye without uniting with those of the opposite side. By this account, the arrangement is different in the monkey alluded to than in man, according to the description of the Wenzels; but it is to be supposed these animals afford great variations in this respect.—(*Journal Complementaire*, October.)

---

### PHYSIOLOGY.

2. *Nature of the Nervous Influence.*—The experiments of MM. Prevost and Dumas render it extremely probable that there exists in every nerve two opposite galvanic currents, and thus serve to explain why the magnetic needle experiences no influence when placed by the side of the nerve at the moment of a violent muscular contraction. Neither is it affected when placed beneath the muscle, or above it, under the same circumstances; and this must depend upon the short distance which separates the ascending and descending filaments of each nervous branch. It remains to explain the contractions produced by the influence of the brain. The authors think that they likewise depend upon galvanic currents; and they have endeavoured to prove this in some circumstances which appeared to them favourable for that purpose. They at first attempted to intercept the current in the pneumogastric nerves; they afterwards placed animals under the influence of the nux vomica, and, during the existence of the tetanus, they endeavoured to ascertain its presence in the different portions of the brain, or in the spinal marrow, or in the sciatic nerves, first whole and then divided. The results have not yet attained a sufficient degree of regularity for publication, but MM. Prevost and Dumas, who are now occupied in these different researches, still hope to be able to satisfy the curiosity of physiologists on this point.—(*Archives generales de Medecine*, September.)

3. ***Obstruction of Blood in the Lungs.***—Dr. Williams relates the following experiments, as proving the cause of the comparative vacuity of the arterial system *post mortem.*

"In one of my examinations, after the animal had been suffocated by making a ligature on the trachea during the acme of inspiration, previous to removing the sternum, I noticed, after the action of the heart had ceased, that the blood still flowed into the right auricle and ventricle, and consequently into the pulmonary artery; and that the propelling agent was so powerful as to distend the right auricle and ventricle so forcibly after the pericardium was slit open, as to make it doubtful whether they would not burst; yet at the same time the pulmonary veins were comparatively empty. In this instance, it was apparent that the blood was obstructed in its course through the lungs, and that this obstruction was one of the principal causes of the vacuity of the circulating system of the arterial blood. From the distention of the cavities of the right side of the heart, and the gorged state of the cavæ, it was evident that no obstacle impeded the return of the blood through the capillaries from the system at large. In a mechanical point of view, the blood ought to have met with equal impediment in passing through the capillaries, as in passing through the final terminations of the pulmonary artery into the pulmonary veins. Impressed with the comparative emptiness of the pulmonary veins, and as no visible subsidence had taken place, I was at a loss how to assign a cause for the obstruction on a mechanical principle. It occurred to me that it was probable that the blood (from its vital principle being exhausted in its route through the system, and from its supply from the thoracic duct being unassimilated,) could not pass from the pulmonary artery into the pulmonary veins without first being acted upon by pure atmospherical air. * * *

"An animal was destroyed by securing the trachea at the acme of inspiration; afterwards the sternum and cartilaginous ends of the ribs were removed. The blood appeared florid in the pulmonary veins, and in the coronary arteries through the pericardium. When the contractions of the left ventricle began to flag, the pulmonary veins became less and less distended, the blood changing from the florid to a darker and darker colour, as the current diminished. At the last contraction the veins flattened, and the left ventricle felt contracted. At this instant an irregular or fluttering contraction of the muscular fibres of the right ventricle commenced, and continued for a short time, excited seemingly by the stimulus of distention, from the accumulation of blood in its cavity. After the irregular muscular action had ceased, the right ventricle felt soft and distended, the left was still contracted, but not so rigid as immediately after the first systole. The pulmonary veins appeared empty: one of them was opened, when only a temporary oozing of blood followed. The pericardium was then slit open, and the right ventricle soon became enormously distended, yet no blood flowed out of the punctured vein. Another pulmonary vein was opened, followed by a similar oozing of blood. The pulmonary artery was now punctured, and instantaneously the blood gushed out, and deluged the shell of the chest.—An animal was examined in the presence of Dr. Traill, after being destroyed in the same manner as the above, and the pulmonary veins were found in the same empty condition after the last systole.

"From the investigation, the following corollaries are drawn:

"1. That the blood is obstructed in its passage through the lungs, on suspension of respiration, while its circulation through the other parts of the body continues.

"2. That the obstruction of the blood in the lungs, on suspension of respiration, is not the effect of a mechanical cause.

"3. That the obstruction of the blood in the lungs, on suspension of respiration, arises from a deprivation of pure atmospherical air.

"4. That the blood which is found post-mortem in the left auricle and ventricle, is the remnant after the last systole, and the subsequent draining of the pulmonary veins.

"5. That the obstruction of the blood in the lungs, on suspension of respiration, is one of the principal causes of the vacuity of the system circulating arterial blood post mortem.

"6. That the immediate cause of the cessation of the action of the heart, is a privation of its natural stimulus, arising from the obstruction of the blood in the lungs. * * *

"Now I flatter myself that the cause of the phenomenon that reserved the discovery of the circulation of the blood to modern times, and to the honour of our country, has been disclosed; and that no one, for the future, however sceptical, will be able to urge the vacuity of the arteries after death as an objection to the doctrine of our immortal Harvey.—(*Annals of Philosophy*, September.)

### PATHOLOGY.

4. *Article on Pathology, in the Encyclopædia Londinensis.*—We have much pleasure in expressing our favourable opinion of a long article on Pathology, which has appeared in the Encyclopædia Londinensis, and to which our attention has lately been directed. It occupies nearly 400 pages, closely printed in double columns; and, although we are willing to admit that in some parts its composition is unequal, and occasionally a few inaccuracies may be selected, still it is, on the whole, a full and comprehensive article. We were much pleased with the introductory matter especially: the account of the foreign schools and universities is replete with interesting information; and there are few, if any, of the modern authors which appear to have escaped the compiler's notice. In the nosological department, after giving a general idea of the different arrangements of the systematic writers, we find Dr. GOOD's classification adopted, but not servilely; for, with regard to the diseases of the skin, the authorities of WILLAN and BATEMAN are chiefly followed. In illustrating the diseases of the skin, we must also give great praise to the engravings, which are taken from Dr. Willan's work: but, in saying this, we must be understood to express ourselves with a reserve, which we really wish to inculcate with respect to all illustrations of medical subjects. That they are beneficial in reviving our recollections, we will not deny; but they must not supersede the study of disease on the living subject. From engravings alone, neither anatomy nor any of the sciences connected with the healing art can be learned; and whoever believes the contrary, is grossly deceiving himself.

We shall dismiss this notice by remarking that the writer of this long article seems intimately acquainted with French medical literature especially, and that he has drawn copiously, yet judiciously, from the writings of LAENNEC, ROSTAN, LALLEMAND, ESQUIROL, &c. &c.

5. *Pustules following the Bite of a Mad Dog.*—M. VILLERMÉ has communicated to the Royal Academy of Medicine the case of a female, whose upper lip was torn by a mad dog. The wound was cauterised at the end of thirteen hours. On the eighth day, a transparent pustule was seen under the left side of the tongue, of the size of a lentil, similar to the pustules observed by M. MAGISTEL under the same circumstances. On the following day this vesicle had disappeared, but was replaced by another, which only lasted twenty-four hours. On the tenth and eleventh days, several other pustules showed themselves; but after that period no others were observed. It is added, that three weeks have elapsed since the bite, but as yet the woman has not shown any signs of hydrophobia.—(*Revue Medicale*, Août.)

6. *Pathology of Mania.*—It is a well-known opinion of M. ESQUIROL that mental alienation often depends upon an anomalous situation of the arch of the colon, and especially upon a vertical position of this intestine: the following case appears to bear upon the point. A woman, aged forty-five, and of a constitution otherwise perfectly healthy, fell into a deep melancholy on the death of her husband, to whom she was tenderly attached. One of the concomitant signs of this melancholy was a great enlargement of the abdomen, accompanied with an oppression of the respiratory organs, and to which succeeded a state of mania, which continued unabated for two years; at the end of which period the patient committed suicide.

On examining the body, besides the ordinary signs exhibited by a body destroyed by hanging, an excessive protrusion of the abdomen was remarked, equal to the size of a woman in the last month of her pregnancy. The head exhibited all the signs of plethora usual upon these occasions. In the thorax there was nothing remarkable. In the abdomen, all the intestines were more or less distended with gas, and showed well-marked signs of inflammation. The stomach, as well as the bladder and uterus, appeared perfectly healthy, whilst the liver and spleen were of an enormous size. But what especially attracted attention, was the total displacement of the transverse arch of the colon, which, with the ascending portion of this intestine, formed a species of triangle.— HUFELAND's *Journal.)*

7. *Case of Elongation of the Uvula, producing Symptoms of Chronic Pneumonia.*—Madame G—, aged thirty, born of healthy parents, had suffered no severe illness from her infancy, when, in the month of January 1822, she became affected with slight cough, to which she at first paid little attention, attributing it to the dampness of the atmosphere and her exposure to cold. This lady was the mother of two children, who were exempt from disease, although of a scrofulous constitution. Nothing announced a predisposition to chronic disease of the respiratory organs: nevertheless, her cough gradually increased till the month of April, when her ordinary medical attendant was consulted. He prescribed various pectoral remedies, without avail: the cough made new progress, and, in the month of August, Madame G. was supposed to be affected with chronic inflammation of the lungs, accompanied with tubercles already developed. Various active means were had recourse to, calculated to retard the progress of the disorganisation.

M. CUYNAT was called in on the 20th of December, 1822, when he observed the following symptoms:—the respiration was constrained, and there existed considerable constriction of the chest; within the cavity of which were felt sharp flying pains, which were increased during the cough and full inspiration. The patient made continual efforts either to swallow or to expel the mucus of the throat; a fixed pain, accompanied with tickling, existed in the larynx; the appetite was almost gone; the tongue was sometimes white, and sometimes natural; the countenance pale; emaciation extreme. The langour both of body and mind of Madame G. seemed to announce the profound lesion of some important organ. The chest, however, examined with much care, was sufficiently sonorous at every part, except the upper, where the sound appeared rather dull. The pulse was sometimes small and unequal, and at others full and frequent; it often passed from one of these states to the other in the space of half an hour. Having examined the fauces, M. Cuynat observed that the uvula was elongated, and rested on the root of the tongue, being the seat of a serous enlargement; and he conceived that all the evils experienced by the patient arose from this cause. He discontinued the use of all internal remedies, and proposed the removal of the superabundant portion of the uvula, as the only means of getting rid of the symptoms. This being agreed upon, the operation was performed on the following day.

The patient was seated opposite a window, with the head supported against the chest of an assistant, and the jaws were kept apart by a roll of linen placed between the molar teeth. The operator then laid hold of the uvula with a pincers, and, pulling it gently, cut off a sufficiently large portion with a blunt-pointed bistoury. No considerable bleeding followed the operation, which was attended with little pain; and the wound was healed under the use of a gargle made of wine and honey, which restored its natural tone to the velum of the palate. The lady entirely recovered.—(*Revue Medicale*, September.)

8. *Obliteration of the Œsophagus, in consequence of Inflammation.* —A young man, aged twenty-four years, was admitted into the Hôtel Dieu at Lyons, having swallowed a glass of aquafortis two years previously. He escaped from the first symptoms, which were violent, but a degree of inflammation remained in the pharynx and œsophagus, which prevented him for a long time from being able to swallow any thing except a little milk and water. The power of swallowing was afterwards lost, and the fluid could only be carried into the stomach by means of a hollow bougie; a still more complete stricture of the œsophagus did not permit even the sound to pass. In this state the patient presented himself to M. Dupuy, on the 18th of May, 1822. The impossibility of introducing food into the stomach induced him to have recourse to nutritive enemata made of soup. The unfortunate patient, tormented by hunger which could not be appeased, was wretchedly emaciated, and affected with violent fever: his eyes were sunk, his tongue red, his belly retracted, and he had continual hiccough. At last, on the 28th, he died of inanition, in the most acute anguish. On opening the body, the œsophagus was found completely obliterated for four inches at its inferior extremity; the stomach was slightly inflamed.—It is suggested that, if it had been possible to have left the sound in the œsophagus, the power of introducing nourishment might have been retained, and the life of the patient prolonged.—*Bulletins de la Société Medicale d'Emulation de Paris*, Juin 1823.)

9. *Doctrines of* M. Lallemand *with respect to Softening of the Brain.*—

1st. There exist resemblances, passing into each other by insensible shades, between the most simple vascular injection of the brain and its membranes, and the most complete apoplexy, (*jusqu'a l'apoplexie foudroyante.*)

2d. The softening of the brain, a disease but little known before the writings of M. Lallemand appeared, has its seat peculiarly where extravasations of blood are most frequent; for example, in the corpus striatum.

3d. The affection results from preceding inflammation. This appears to be proved, according to M. Lallemand, by the red circle,—by the injection of blood, or the infiltration of pus, which surround the softened portions,—by the causes which have operated,—and by the symptoms of irritation which show themselves in the first instance.

4th. M. Lallemand appears at first to have limited his remarks on softening as a result of inflammation to the brain, but since then his ideas have extended, and he now attributes softening of all the tissues to the same cause. He has shown that, although the inflamed organs are hard and very dense, yet their tissue has in truth but little resistance, and that the slightest force disunites their texture.

5th. The softening sometimes precedes apoplexy, and sometimes follows it: it is sometimes the cause, and sometimes the effect; but in every case it is the product of inflammation.

6th. Apoplexy is preceded by softening of the brain, as all hemorrhagies by the inflammatory irritation of the tissues where they occur. It is correct to say that hemorrhagy often proves abortive, *(l'hemorrhogie avorte,)* and then there is only inflammation for the organs: that is to say, softening of the brain, in the case under consideration.

7th. If the softening of the brain affects the grey substance more frequently than the cuticle, it arises from the former having a more extended surface, from its receiving larger and more numerous blood-vessels, and likewise from the arachnoid which surrounds it often communicating the inflammation by which it is affected.

8th. In apoplexy there is sudden paralysis, but in inflammation of the arachnoid there are convulsions; and afterwards palsy, when the brain becomes inflamed.

9th. The following are the circumstances which generally occur in apoplexy: at first there is hemicrania, head-aches, giddiness; then pains, prickings, and cramps; creeping in the limbs next come on; and then melancholy and moroseness. It is then that inflammation of the brain exists. If symptoms of palsy appear, softening of the organ may be suspected. Finally, if a deposition of blood takes place in the brain, then the paralysis is complete, and the apoplexy evident. After some time, if the attack has been slight, fresh pains come on, which are the signs of a new inflammation developed around the effused clot: it is this same inflammation which hastens the reabsorption of the blood and the formation of a cicatrix, and which likewise may occasion a fresh attack, by the softening which it produces.

10th. Delirium and convulsions announce inflammation of the arachnoid membrane; while drowsiness, coma, and paralysis, denote inflammation of the brain.

11th. The palsy of softening may not be preceded by inflammatory symptoms; but then it is always more gentle than the palsy caused by apoplexy.

12th. Every time that the brain becomes diseased when it is exposed and in contact with the atmospheric air, it swells, and a fungus protrudes through the cranium: this is an effect of the movements and inflammatory action of the brain. After death, in place of a fungus, we only find a fissure—a large cavity.

13th. If, in the case of inflammation of the brain, there be no external wound, then the affected part of one hemisphere compresses the neighbouring parts of the opposite side: hence arise the drowsiness, coma, and loss of sense,—deafness and blindness. If there be an external opening, then the palsy and loss of sense only happen on the side opposite the inflammation; but consciousness is retained, because the integrity of a single hemisphere suffices for the exercise of the intellect.

---

### THERAPEUTICS.

10. *Amenorrhœa treated by Injecting a Solution of Ammonia.*—The following extract we give, without comment, from a paper by FRANCOIS LAVAGNA, of whom we know nothing further than that he is a doctor in medicine, and a correspondent of the "Journal Universel des Sciences Medicales."

"There is no physician who does not frequently, in the course of his practice, however limited, see young girls, about the age of puberty, who are pale, languishing, and irregular with respect to menstruation, recover their ruddiness, and assume new vigour, under the influence of periodical evacuations of the catamenia supervening upon marriage. This fact had induced me to think that any method capable of determining a flow of blood towards the uterine system, would be as well calculated as coition to effect a return of the menses, when suppressed or retarded; when I conceived the resolution of making some experiments on this subject with ammonia. This medicine, of which the stimulation is very transitory, when it is suspended in luke-warm milk, or in any other liquid, ought to be injected into the cavity of the uterus, or at least into the vagina, in sufficient quantity to excite in these parts the orgasm which the act of generation produces in them, *(a une dose suffisante pour exciter, dans ces parties, l'orgasme que produisent en elles l'acte generateur !)*"!

Various cases are detailed in which the remedy, thus employed, proved successful; and the author gravely informs us, that the young ladies thought the excitement thus produced "*une sensation desagreable.*"—*(Journal Universel des Sciences Medicales*, 93 *Cahier.)*

11. *Chlorine, with Water, employed in the Treatment of Scarlatina.*—Dr. BRAUN, who details the advantages of this remedy, carries his enthusiasm so far as to call it a specific in scarlet fever. During ten years he has employed it successfully in the most complicated cases. Chlorine mixed with water, he says, destroys contagion; and the cure is seldom followed by any consecutive disease. He gives this medicine in the dose of a tea-spoonful every two or three hours, to children from three to six years of age, and, to those of a more advanced age, a table-spoonful in the same intervals. It is given without the addition of any

525

### Captn. Jekyll's Patent Vapour Bath.

*The Cloak is made of Oil'd Silk, which does not expose the Person: as it is here represented to shew the Apparatus of the Bath.*

other medium, as the chlorine is decomposed in most mixtures. It should be swallowed quickly, lest it should produce cough. M. Braun observes, that, in the sore-throat which often accompanies the scarlet fever, this water is more easily swallowed even than mucilaginous drinks. As soon as the disease begins to decline, he only prescribes the medicine in the dose of one or two spoonsful in the day. The whole quantity taken during the disease has never exceeded two ounces in the cases of children, and four or five ounces in those of adults. If this medicine is given in greater doses, it brings on vomiting and frequent alvine evacuations: if it is not fresh, or has been exposed to the atmospheric air, it produces slight excoriations of the lips. M. HUFELAND had already recommended this remedy in nervous and typhus fevers.—(*Revue Medicale*, Juillet.)

### MIDWIFERY.

12. *Abortion induced by thrusting a Seton-needle into the Uterus.*—M. CROUZIT was called, in 18—, to a girl, who was pregnant and laboured under uterine hemorrhage. Various measures had been employed without the *desired* effect; when, at length, recourse was had to the introduction of a seton-needle into the uterus. The instrument was thrust in so far that it became impossible to retain the hold of it, and, of course, it could not be withdrawn. The imprudent operator quieted the apprehensions of his patient, by assuring her that it would come away with the fœtus,—and disappeared. When M. Crouzit arrived, the ovum had been expelled, and appeared about three months old, but the after-birth was retained. It was impossible to ascertain the exact situation of the needle; nor could the placenta be removed, in consequence of the contraction of the neck of the uterus: on examining through the parietes of the abdomen, the surgeon thought he could discover the foreign body. After two days the secundines came away without the needle, and it seemed probable that, being introduced by its sharp end, this instrument had pierced through the uterus during its contraction, and successively traversed various parts, as it was not until eleven days after the event that the patient began to experience pain in the groin. On the thirty-fifth day, an elevated point was perceived in this situation, and the pain became very acute. On the seventy-ninth day, the instrument was withdrawn, by the patient herself, from this part. It proved to be a seton-needle,—a stylet of silver, six inches (French) in length, buttoned at one extremity, and sharp at the other. The wound speedily closed, and no inconvenience was experienced from the occurrence!!!

### CHEMISTRY.

13. *Economical Method of making Prussic Acid.*—M. PESSINA, of Milan, gives the following process for the formation of the prussic acid, which he states to be by much the most economical hitherto discovered.—Eighteen parts of the prussiate of iron and potass are to be reduced to fine powder, and introduced into the bulb of a small tubulated retort of glass, to which a small balloon, likewise tubulated, is then to be attached. A conducting tube dips into a flask containing a small quantity of distilled water. A *cold* mixture, of nine parts of sulphuric acid with twelve of water, is next introduced into the retort, which is closed, and the whole left for twelve hours; the balloon, and the neck of the retort, being kept cool with ice and evaporating cloths. A gentle heat is then applied, and continued until the striæ in the neck of the retort become more rare, and a blue substance rises, as if it would pass into the receiver. The heat is now to be withdrawn, and the apparatus allowed to cool; the contents of the receiver being preserved in a proper vessel. The prussic acid thus obtained is stated to be pure, and the specific gravity about 0.898.—(*Giornale di Fisica.*)

### NATURAL HISTORY.

14. *On the Metamorphoses of certain Species of Confervæ.*—At a late meeting of the Royal Academy of Sciences, MM. Bosc, Dumeril, and Savigny, made a Report on a Memoir presented by M. Gaillon, the subject of which is the Metamorphosis of certain Species of Confervæ into inferior Animals. Many naturalists, and lately M. Berg de St. Vincent, have stated that a great number of the confervæ become disorganised during the summer, and the greenish globules seen in their inside become animalculæ, which swim for some time in water, are capable of being irritated by the touch, and terminate their career by again forming fresh confervæ. These singular beings alternately destroy, in a remarkable and unequivocal manner, the feeble limits established between the two kingdoms. M. Gaillon presents some facts in his memoir which are perfectly in accordance with those already known. The species of conferva which he has particularly examined belongs to the genus *Ceromion* of De Candolle. Delwyn has described and represented it under the name of *conferva comoides*, it covers the border of the sea at Dieppe. During a whole year M. Gaillon has observed, at certain times, greenish, egg-shaped, little bodies detach themselves spontaneously from the filaments of the *conferva comoides*, advancing sometimes rapidly, sometimes slowly,—changing their direction,—in fact, acting in the same manner as the Enchelier, the Cyclides, and the other infusory animals of Muller. Taking the entire filament, he has forced these animalculæ to detach themselves before their time, and has observed the same phenomena. M. Gaillon supports his opinion by the authority of M. Borg de St. Vincent, and also of M. Mertans, the celebrated German botanist, who writes that he is not surprised at their observations, having for a long time conceived the same idea. Last year (he says,) I showed to a number of sçavans the *conferva mutablis*, which, on the 3d of August, resolved itself into molecules,—which on the 5th were endowed with motion,—on the 6th were re-united in the form of a simple articulation,—and resumed, on the 11th, its primitive form.—(*Archives Gen.* Sept.)

In an early Number of the "Philosophical Magazine," there is an account of some microscopic observations, made by M. Coquebert, on the *conferva jugalis*; by which it appears that the male and female filaments actually unite,—that certain globules contained in the male filaments actually pass into the interior of the female filaments,—and that, by this union, there are formed in the latter seeds, or ova, which re-produce the species.

### MISCELLANEOUS.

15. *Vapour Bath.*—The *Plate*, which we have given in this Number, is the representation of a vapour bath, the invention of Capt. JEKYLL, of the Navy, whose name has long been familiar with the public for his many improvements in mechanism. His own case drew his attention to the subject of vapour-bathing, and the result of his application has been the invention of the annexed admirable portable-bath, which has many advantages over those that have hitherto been in use. But, as vapour bathing is by no means to be used without discrimination, we purpose entering more fully on the subject in a subsequent Number, resting for the present with advising that it should only be resorted to under the direction of a medical man.

The construction of this bath will readily be understood by merely examining the plate, as the oil-skin dress is represented transparent in order to show the mechanism. The boiler is provided with a safety-valve; the tube that conveys the steam passes into a round box placed beneath the patient's feet, called the *dispenser*, and, by means of handles, (one of which is seen in the patient's hands,) the cocks are turned, by which the steam is suffered to escape. The dispenser is cup-shaped, so that any medicament that may be thought proper can be introduced into it. There is also a contrivance, not here exhibited, by which the vapour may be applied to a limb, to the ear, or to a solitary enlarged gland, if necessary.

16. *Facts of the Case of* Mr. MOREL, *Surgeon to the Forces.*—In a "brief memoir" of the professional character of the late Mr. Morel, contained in the Transactions of the Associated Apothecaries, the following note occurs:

"He was on the staff twenty years all but a few months: had he been allowed to remain there the proper time, he would have retired upon full-pay, which his active, arduous, and zealous, services justly entitled him to. But he was *obliged* to retire prematurely upon half-pay, having been informed that the hospital establishment was immediately to be broken up. Instead of which, the situation which he was thus induced to relinquish was given to another, and the hospital not broken up till after the time had expired which would have determined his rightful claims. He felt this unfair treatment most severely, and, added to other professional mortifications, those injuries sunk deep into his mind, and injured his health."

Anxious, as one of its members, to vindicate the character of the medical department of the army, as well as influenced by a sense of justice, we take this early opportunity of stating that the paragraph given above is an entire misrepresentation. We have made it our business to ascertain the truth, and our readers may rest assured of the accuracy of the following statement: how well it agrees with that of his "colleague," will be seen by comparing them.

Mr. Morel had served on full pay *only sixteen years*. Had he completed a service of twenty years, he would not have been entitled to full pay; nor to any thing beyond the half-pay of 7s. a-day, (which rate he received,) unless the cause of reduction were ill health, when he might have claimed 10s. a-day. The reduced establishment of medical officers required that all those who were retained on full-pay should be fit for any service; but Mr. Morel was much afflicted, and was, in fact, scarcely equal to his share of the duties that then devolved on the limited number of officers retained at the York Hospital. In consideration of his broken-down constitution, the heads of his department made strenuous efforts with the Secretary at War and Board of Treasury, to obtain for him a pension of 100*l.* a-year: they had to regret the want of success in their application; but Mr. Morel was very sensible of, and expressed his gratitude for, their exertions. On his retirement, he obtained the brevet rank of Deputy Inspector of Hospitals, as an honourable mark of approbation of his conduct as a medical officer.

17. Mr. COPLAND HUTCHISON's *Dismissal from the Penitentiary.* —The minutes of the evidence before the committee of the House of Commons, on the subject of the Penitentiary at Millbank, extends to no less than 399 folio pages: much of this we have toiled through, but, such is the conflicting nature of the testimony upon the medical points, that we confess ourselves unable to come to any satisfactory conclusion; nor, indeed, do we think that the evidence before us affords sufficient data to satisfy the judgment of an impartial inquirer. It is not, therefore, our intention to enter upon this question at present; but there is another point, which we cannot allow ourselves to pass over in silence, open as the pages of our Journal are, and we trust will ever be, to support the dignity and honour of the medical profession, by vindicating the character of its members. It is impossible to read the voluminous papers before us without feeling convinced that the charges against Mr. Hutchison were of the most vexatious kind: not directed against his professional talents,—not against his zeal and attention,—not against his humanity and kindness, (for on all these points he was invulnerable,) —but an attempt was made to prove that, on one solitary occasion, he had made his evening visit in a state of inebriety; and that he evinced a general indisposition to communicate with the superior officers of the establishment. The first of these charges was fully disproved before the committee of the House of Commons,—not merely as regards the general habits of Mr. H., but even with respect to the particular evening in question: while, with regard to the second, there is not the slightest evidence whatever of the disinclination to open and free communication having existed on *his* part. The following is the statement of the committee of the House of Commons:

"Your committee have also inquired into the causes of the dismissal of the late medical superintendant, as well as into the accusations preferred against him in May and June, 1822. On the first part of the subject, your committee think it their duty to observe, that the testimony of all the members of the managing committee, who have been examined, speak most favourably of the skill, kindness, and attention, of that officer: he has received also, from persons of the highest rank in his profession, testimonials of character the most flattering; yet, at the same time, there does appear to have arisen between him and the managers of the establishment differences, which, for the good of the institution, rendered his continuance in the situation he filled inexpedient. Your committee give no opinion on the cause of these differences; but the mere fact of their existence is to them a sufficient proof that the parties could not go on together amicably, or with that cordial union and confidence which their respective situations, of necessity, demand."

When it is considered that Mr. Hutchison acted gratuitously for three years and a half; and that, at the end of that time, being offered 300*l.* a-year for his services, he accepted only of 200*l.*; and that, during seven years' constant attendance, his conduct had, in the words of the committee, been remarkable for "skill, kindness, and attention;" we cannot but regret that skill, kindness, and attention, should have been so rewarded. Mr. Hutchison, it is to be remembered, was dismissed by the committee of the Penitentiary, before the parliamentary inquiry into his conduct; and that his acquittal by the committee of the House of Commons thus becomes unavailing, except as regards the vindication of his character. With regard to his professional character, as influenced by these proceedings, we beg to give the following note:

"*Whitehall; March* 15, 1823.

"Sir,—I am directed by Mr. Secretary Peel to acknowledge the receipt of your letter of this day's date, in which you request that he will either lay the correspondence therein inclosed before the House of Commons, or take some other means of vindicating your professional character to that House. And Mr. Peel desires me to acquaint you in reply, that nothing which has passed in the House of Commons tended to throw the slightest imputation upon your professional character; nor has any communication been made to him, directly or indirectly, imputing the present disorder in the Penitentiary to your want of skill or attention.

"I have the honour to be, Sir, your most obedient humble servant,

"H. HOBHOUSE."

That all the members of the committee of the Penitentiary did not coincide in the measures adopted, will appear from the following extract of a letter from Mr. WM. MORTON PITT:

"Feeling as Dr. Hutchison has felt, and still feels, I cannot discover any thing objectionable in his expression of those feelings, or in the *tone* of that letter, which has caused him to be dismissed from his situation; and, judging from what I have myself observed of his attention to the performance of his duties, and from his character for so many years, I cannot, consistently with what I conceive to be the truth, disguise the opinion I have formed, that unfortunate circumstances have caused steps to be taken, which grievously, and I must say that it appears to me undeservedly, do injure his character."

—and, in another place, "I certainly am of opinion that Dr. Hutchison has received *harsh treatment and undeserved injury.*"

In quoting these expressions, we do but echo the sentiments of all unprejudiced persons who have taken the trouble to inquire into the facts; and, while we believe that those gentlemen who were appointed in his place have no very enviable situation, we confidently trust that the government will not permit an old and zealous servant to suffer for charges of which he has been acquitted by the Parliament.

18. *A Catalogue of the detached Medical Papers published by the late* Dr. M. BAILLIE.—Since our Biographical Sketch of Dr. Baillie, we have been enabled to procure a list of his papers, which we believe will be found correct.

1. An Account of a remarkable Transposition of the Viscera in the Human Body.—*Philosophical Transactions for* 1788.
2. An Account of a particular Change of Structure in the Human Ovarium.—*Philosophical Transactions for* 1789.
3. On the Want of a Pericardium in the Human Body. 1788.—*Transactions of a Society for the Improvement of Medical and Chirurgical Knowledge*, vol. i. p. 91.

4. Of a remarkable Deviation from the natural Structure in the Urinary Bladder and Organs of Generation of a Male. 1790.—*Ditto*, vol. i. p. 189.

5. A Case of Emphysema, not proceeding from local Injury. 1791.—*Ditto*, vol. i. p. 202.

6. An Account of a Case of Diabetes, with an Examination of the Appearances after Death. 1795.—*Ditto*, vol. ii. p. 70.

7. An Account of a singular Disease in the great Intestines. 1796.—*Ditto*, vol. ii. p. 144.

8. An Account of the Case of a Man who had no Evacuation from the Bowels for nearly fifteen Weeks before his Death. 1797.—*Ditto*, vol. ii. p. 174.

9. Remarks on Schirrous Structures, in a Letter to Dr. ADAMS, dated Nov. 10, 1796.—Published in Adams's *Observations on the Cancerous Breast*, 1801, p. 32.

10. On the Embalming of dead Bodies. 1804.—*Transactions of a Society for the Improvement of Medical and Chirurgical Knowledge*, vol. iii. p. 7.

11. An Account of several Persons in the same Family being twice affected with Measles. 1808.—*Ditto*, vol. iii. p. 258.

12. Additional Instances of Measles occurring twice in the same Person. 1810. *Ditto*, vol. iii. p. 263.

13. Three Cases of Inflammation of the inner Membrane of the Larynx and Trachea, terminating quickly in Death. 1809.—*Ditto*, vol. iii. p. 275.

14. The Case of a Boy, seven Years of Age, who had Hydrocephalus, in whom some of the Bones of the Skull, once firmly united, were, in the Progress of the Disease, separated to a considerable Distance from each other. 1806.—*College Transactions*, vol. iv. p. 1.

15. Of some uncommon Symptoms which occurred in a Case of Hydrocephalus Internus. 1806.—*Ditto*, vol. iv. p. 9.

16. Upon a strong Pulsation of the Aorta in the Epigastric Region. 1812.—*Ditto*, vol. iv. p. 271.

17. Upon a Case of Stricture of the Rectum, produced by a spasmodic Contraction of the internal and external Sphincter of the Anus. 1814.—*Ditto*, vol. v. p. 136.

18. Some Observations respecting the Green Jaundice. 1814.—*Ditto*, vol. v. p. 143.

19. Some Observations on a particular Species of Purging. 1814.—*Ditto*, vol. v. p. 166.

20. Some Observations upon Paraplegia in Adults. 1817.—*Do.* vol. vi. p. 16.

19. *Chloride of Lime applied to counteract Putrefaction.*—MM. ORFILA, HENNELLE, GERDY, and LESECEUR, give an account of the examination of the body of a person named Bourcier, which had been buried a month in the cemetery of Père la Chaise. The exhumation took place by order of the legal authorities, in consequence of a suspicion of the person having died by poison. On disinterring the body, it exhaled an infectious odour, and remained three hours in the open air previous to the arrival of those persons who were to prove its identity; it was then moved into a large and well-aired situation. The odour had by this time become much more intolerable, and the body had swelled in a very perceptible manner, and, by the recommendation of M. LABARRAQUE, it was sprinkled with the chloride of lime dissolved in water; the effect of which was wonderful. After a few aspersions, the infectious odour was instantly destroyed, and it became possible to proceed to the examination. We pass over the processes of the examination, and merely state, that a quantity of fluid found in the abdomen and thorax was collected with a clean sponge, and put into a bottle; and the pharynx, stomach, and the whole intestinal canal, were taken out, and put into alcohol; and, upon examination, decided proofs of the presence of the white oxyde of arsenic were found in the intestines, both large and small.—(*Journal des Sciences Univ.* Août.)

### METEOROLOGICAL JOURNAL,

*From October 20, to November 19, 1823.*

By Messrs. WILLIAM HARRIS and Co. 50, Holborn, London.

| | MOON. | Rain gauge | THERM. | | | BAROM. | | DE LUC'S HYG. | | WIND. | | ATMOSPHERIC VARIATION. | | |
|---|---|---|---|---|---|---|---|---|---|---|---|---|---|---|
| | | | 9 A.M. | MAX. | MIN. | 9 A.M. | 10P.M. | 9 A.M. | 10P.M. | 9 A.M. | 10P.M. | 9 A.M. | 2 P.M. | 10P.M. |
| Oct. | | | | | | | | | | | | | | |
| 20 | | .57 | 55 | 57 | 50 | 29.85 | 30.00 | 87 | 84 | ENE | ENE | Cloud. | Fine | Fine |
| 21 | | | 53 | 56 | 50 | 30.11 | 30.11 | 83 | 80 | NE | NNE | Fair | | |
| 22 | | | 52 | 58 | 50 | 30.05 | 29.90 | 75 | 75 | ENE | NNE | | | |
| 23 | | | 50 | 56 | 52 | 29.90 | 29.85 | 70 | 67 | ENE | NNE | Fine | | |
| 24 | | | 50 | 55 | 48 | 29.90 | 30.00 | 72 | 75 | ENE | NE | | | |
| 25 | ☾ | | 45 | 55 | 45 | 30.16 | 30.25 | 77 | 76 | NE | N | | | Foggy |
| 26 | | | 42 | 48 | 44 | 30.33 | 30.25 | 78 | 80 | N | W | Foggy | Overc. | Overc. |
| 27 | | | 45 | 46 | 45 | 30.14 | 30.00 | 81 | 87 | W | WSW | | | Foggy |
| 28 | | | 50 | 53 | 46 | 29.74 | 29.47 | 85 | 89 | SSW | SE | Overc. | Cloud. | Rain |
| 29 | | | 49 | 52 | 44 | 29.55 | 29.55 | 80 | 85 | WSW | SSW | Fine | Fine | Fine |
| 30 | | | 42 | 45 | 44 | 29.24 | 29.04 | 90 | 90 | NE | NNE | Rain | Rain | Rain |
| 31 | | .97 / 1.54 | 41 | 44 | 39 | 28.93 | 29.25 | 91 | 90 | NNW | N | Rain | Storm. | Storm. |
| Nov | | | | | | | | | | | | | | |
| 1 | | | 40 | 42 | 34 | 29.60 | 29.83 | 87 | 85 | NNW | NNW | Fair | Fine | Fine |
| 2 | ● | | 39 | 43 | 33 | 30.00 | 30.00 | 84 | 87 | NNW | NNW | Fine | Fine | Fine |
| 3 | | | 38 | 43 | 45 | 29.90 | 29.75 | 85 | 88 | WSW | SSW | Foggy | | Rain |
| 4 | | | 50 | 51 | 44 | 29.57 | 29.46 | 87 | 89 | S | SE | Fine | | Cloud. |
| 5 | | | 45 | 51 | 45 | 29.56 | 29.60 | 90 | 90 | E | ESE | Rain | Rain | Rain |
| 6 | | | 51 | 52 | 49 | 29.80 | 29.79 | 90 | 89 | E | ENE | Foggy | | Cloud. |
| 7 | | | 50 | 54 | 49 | 29.84 | 29.96 | 90 | 90 | N | NNW | Rain | Cloud. | |
| 8 | | .73 | 51 | 51 | 40 | 30.30 | 30.30 | 84 | 80 | ENE | ENE | Fine | Fine | Fine |
| 9 | | | 42 | 50 | 39 | 30.38 | 30.46 | 70 | 67 | E | NE | Fine | Fine | |
| 10 | ☽ | | 41 | 41 | 28 | 30.47 | 30.40 | 65 | 67 | E | ENE | Foggy | Foggy | Foggy |
| 11 | | | 32 | 35 | 30 | 30.32 | 30.30 | 79 | 79 | ENE | SE | | | Fine |
| 12 | | | 35 | 40 | 34 | 30.30 | 30.30 | 75 | 78 | SE | SW | | | Foggy |
| 13 | | | 37 | 39 | 35 | 30.17 | 30.17 | 80 | 84 | SSW | SW | | Foggy | |
| 14 | | | 34 | 37 | 34 | 30.14 | 30.10 | 80 | 80 | SW | SW | | Fine | Foggy |
| 15 | | | 46 | 49 | 44 | 30.13 | 30.23 | 84 | 80 | W | N | Overc. | Cloud. | |
| 16 | | | 46 | 46 | 40 | 30.30 | 30.25 | 78 | 80 | N | N | Fine | Foggy | Foggy |
| 17 | | | 38 | 41 | 38 | 30.25 | 30.23 | 88 | 85 | N | N | Misty | Cloud. | Cloud. |
| 18 | ○ | | 49 | 49 | 37 | 33.30 | 30.27 | 80 | 78 | N | E | | | |
| 19 | | | 44 | 47 | 44 | 30.10 | 30.00 | 70 | 75 | E | SW | Cloud. | Overc. | Fair |

The quantity of rain fallen in the month of October, was 1 inch and 54.100ths.

## Figures and Tables

**Figure f1:**