# Errata

**Published:** 1824-01

**Authors:** 


					i . d28, tor chloride ot lime, read chloruret of June.

				

